# Access and barriers to reproductive mental health services. A mixed-methods examination of self-stigmatization, help-seeking motivation and experiences with primary or reproductive healthcare professionals

**DOI:** 10.3389/fgwh.2025.1658534

**Published:** 2026-01-05

**Authors:** Luisa Fornasiero, Anna-Lena Horn, Katrin Braune-Krickau, Fabienne Forster

**Affiliations:** 1 Psychological Institute, Zurich University of Applied Sciences, Zurich, Switzerland; 2 Department of Gynecopsychiatry, Psychiatry St. Gallen, St. Gallen, Switzerland

**Keywords:** self-stigma, help-seeking, reproductive mental health, peripartum mental health, psychotherapy

## Abstract

**Objective:**

Specialized reproductive mental health services help to alleviate symptoms of mental disorders associated with the reproductive system such as menstrual cycle, infertility, pregnancy or birth. However, people by reproductive mental disorders often do not receive the treatment they need or treatment is initiated after a long delay. The current study examined the process of accessing specialized reproductive mental health services in patients (mostly cisgender women) who just attended their first psychotherapy session in these services. Specifically, we examined the role of self-stigmatization related to mental issues as well as experiences with non-mental healthcare professionals and expectations regarding reproductive mental health services.

**Method:**

The sample consisted of 106 cisgender female and 3 cisgender male patients who recently attended the specialized reproductive mental health service (called gynecospsychiatry). Data were collected using a mixed-methods design based on validated questionnaires and open-ended text questions in an online survey. Additionally, clinical information was obtained from the treating psychotherapists. T-tests and regression analyses were applied to quantitative data. Open ended questions were analyzed qualitatively using thematic analysis.

**Results:**

Quantitatively, self-stigma was not significantly associated with the process of help seeking. Furthermore, our results suggest that merely being approached by primary or reproductive healthcare professionals was not associated with lower levels of self-stigma in our sample. Qualitative-analyses showed two major themes: 1) Not beating around the bush – clear words instead of overlooking or downplaying psychological distress, 2) Wanting to feel understood and accepted as opposed to condemned, judged, and devalued.

**Conclusion:**

Based on these findings, recommendations were made for psychotherapists as well as primary or reproductive healthcare professionals about how those affected by reproductive mental disorders can best be supported to seek treatment. Further research is recommended with larger clinical samples of patients with reproductive mental disorders.

## 1 Introduction

The current study examines a sample of patients with reproductive mental disorders, who just attended their first session with psychotherapist (psychologist or psychiatrist) specialized in reproductive mental health services. These outpatient services called “gynecopsychiatry” are embedded in the outpatient clinic within a general psychiatric hospital and are specialized in mental disorders concerning the menstrual cycle or within the context of infertility, pregnancy, birth and the first year postpartum. Reproductive mental disorders are common (1) and are associated with the well-being of not only the patients but also their children, partners and social environment (2–4). Unfortunately, a large proportion of people affected by reproductive mental disorders remain without treatment for too long (5, 6). Therefore, it is important to identify the factors that prevent individuals from seeking treatment so that strategies for reducing these barriers can be developed. The current study examined factors that complicated or prolonged the process of getting mental healthcare in patients who did get treatment. Specifically, the role of self-stigmatization due to mental issues as well as deterring and supportive experiences with primary or reproductive healthcare professionals (such as gynecologists, midwifes and general practitioners) and expectations of specialized reproductive mental health services were examined using a mixed-methods approach combining quantitative and qualitative analyses.

There are three main groups of patients (mostly cisgender women) attending specialized services for reproductive mental health. Most patients attending the reproductive mental health services are affected by mental disorders during pregnancy and the first year postpartum. The peripartum period carries a high risk of serious mental disorders with one in four parents affected by some kind of mental disorders (7, 8). Most common disorders are affective and anxiety disorders with prevalences of about 15%–20% in both disorders (9). A second group of patients attending the reproductive mental health services are couples struggling with infertility. About half of all women with infertility are affected by adjustment disorders (10–12). In men and women, infertility is positively associated with mood disorders, anxiety and depressive symptoms (13, 14). The third group consists of patients (mostly cisgender women) with mental health issues related to the menstrual cycle. Existing mental disorders such as psychotic, panic, eating, affective and borderline personality disorders were shown to exacerbate during the perimenstrual phase (15). Furthermore, there is a high comorbidity of around 40%–50% between mood disorders and premenstrual disorders such as the premenstrual syndrome and premenstrual dysphoric disorders (16). Premenstrual symptoms during the luteal phase that fulfil the criteria for premenstrual dysphoric disorder (PMDD) are prevalent in around 3.2% of cisgender women in reproductive age (17). Additionally, around half of all menstruating cisgender women experience various emotional and physical symptoms in the luteal phase, which are experienced as distressing and impairing the quality of life in around 20% of menstruating women (18, 19).

Specialized reproductive mental health services are characterized by their expertise in endocrinological, mental, physical and social aspects of distress and disorders associated with the reproductive system. While phenomenology and risk factors for peripartum and reproductive mental disorders are largely similar to those for mental disorders in general, treatment considerations may differ. For example, pregnancy and breastfeeding influence the availability of different treatment options (20) and present diagnostic challenges to clinicians (21). In individuals with infertility, specific psychological interventions were found to be beneficial for mental health and pregnancy rates in a recent meta-analysis (22). Furthermore, newer studies indicate a need for more personalized clinical approaches that integrate menstrual cycle dynamics into psychotherapy (23, 24).

Hence, psychotherapists need specialized knowledge when treating reproductive mental disorders. In Switzerland, such a specialized service can be found in the reproductive mental health called gynecopsychiatry (25). This specialized psychotherapy service treats patients who from mental conditions related to gynecological issues such as peripartum mental illness, infertility, premenstrual mental disorders or traumatic experiences during pregnancy or childbirth.

While the specialized service of reproductive mental health services is well used, the demand is still not remotely as large as prevalences would indicate. However, timely and adequate psychotherapy is important since reproductive mental disorders do not only have negative consequences for those directly affected but can also have a negative impact on the parent-child attachment (26). Specialized treatment at an early stage of illness can counteract the exacerbation of symptoms (27). Therefore, the current study aims to detect factors that may explain why many of those affected do not seek psychotherapeutic support or not early enough (5, 6).

One potential explanation for why people experience difficulties in seeking therapy resides in the presence of stigma. Stigma can be divided into two forms: public stigma and self-stigma (28). Public stigma occurs when elements of labelling, stereotyping, linguistic demarcation and power asymmetry come together (29). If public stigma is internalized and directed against oneself, this is known as self-stigma (30). The degree of self-stigma experienced by an individual is influenced by the extent of their exposure to public stigma (31). People with mental illness who have internalized the public stigma may adopt self-stigmatizing beliefs (32), which can significantly reduce the willingness to seek therapy, as they may fear judgment or believe they are undeserving of help (33). Stigma associated with mental health conditions can lead to delayed or avoided therapy-seeking behavior (34–36). In paripartum women, a review of 28 studies found a generally low intention to seek help for mental health problems and high levels of stigmatizing attitudes towards peripartum mental health problems in the public (37).

A key factor for reducing stigma around mental disorders is the professional handling of mental health issues (38, 39). Support from healthcare professionals can be positively associated with treatment utilization (67, 68). In the context of reproductive and peripartum mental health, multiple health care professionales are typically involved, including general practitioners (GP), gynecologists, midwives and parenting counsellors. These professionals represent critical points of contact for mental health promotion (40). When properly trained, these professionals can contribute to reducing self-stigma and promoting treatment uptake (41, 69, 70).

However, such training is often lacking in primary or reproductive healthcare professionals. For example, many midwives report a lack of peripartum mental health knowledge, training, and confidence, which was associated with a lower engagement in mental health promotion (41). Among postpartum nurses, perceptions of inadequacy and difficulty of taking responsibility for women with mental illness are also commonly reported (42). Similar results are found in healthcare providers concerning reproductive and sexual health (43). Based on our clinical experience, patients were often informed about the specialized services of reproductive mental health services by midwives or parenting counsellors. However, evidence of their impact on self-stigma and help-seeking is lacking. Accordingly, this study examines the role of different professionals involved in reproductive and peripartum health.

A third factor that may influence whether potential patients initiate psychotherapy may be expectations concerning psychotherapy and mental health services in general. Studies consistently show that the general public has relatively poor knowledge of mental health symptoms and treatment (44). Similarly, a large proportion of women in reproductive age showed a poor mental health literacy, which was identified as a major obstacle for help-seeking (45). On the other hand, more knowledge about mental health was positively associated with identifying mental health problems (46). Furthermore, assuming that therapy might help was reported to be a key motivator in the process of seeking psychotherapy (47).

This study aims to enhance our understanding of the factors influencing individuals' willingness to engage with reproductive mental health services when facing reproductive mental disorders. Specifically, it was hypothesized that a higher level of self-stigma is associated with a longer time for each of the four steps of help-seeking: recognizing the problem (H1a), considering psychotherapy as a potential solution (H1b), deciding to seek psychotherapy (H1c) and scheduling an appointment (H1d). Furthermore, we expected a significant difference in self-stigma between individuals who were approached regarding their mental health issues by involved professionals and those who were not (H2). Additionally, we examined which professionals discussed mental health with patients and patients' preferred professionals for such discussions. The qualitative part of the study explored helpful or hindering experiences with primary or reproductive healthcare professionals as well as encouraging or discouraging expectations of patients toward reproductive mental health services.

## 2 Materials and methods

### 2.1 Setting

The data was collected using a mixed-methods design. All participants were patients of reproductive mental health services in the Swiss canton of St. Gallen, which treats people with mental illnesses that meet at least one of the following criteria: a) psychiatric disorders during pregnancy and in the postpartum period up to the end of the first year (pre-existing or newly diagnosed); b) psychopharmacotherapy during pregnancy and lactation; c) pre-conception assessment for individuals with psychiatric disorders wishing to conceive; d) traumatic birth experiences; e) adversities in the peripartum period; f) miscarriage, stillbirth/neonatal death, congenital abnormalities, disabilities of the child; g) unfulfilled desire for children. This outpatient services are part of the public health care services of the region and thereby partially subsidized by the local government (canton of St. Gallen).

### 2.2 Ethics and governance

The current data set is part of a larger study which was reviewed and approved by the regional ethics committee of eastern Switzerland (EKOS) (BASEC No. 2023-01260). The present observational study was preregistered on the Open Science Framework OSF (Registration DOI: https://doi.org/10.17605/OSF.IO/85XUW).

### 2.3 Sample

All patients attending reproductive mental health services from October 2023 to December 2024 who had sufficient German language skills and to answer the online questionnaires were invited to participate in the study. Potential participants received a study flyer from their therapist within the first week after their first consultation. A total of 106 cisgender women and 3 cisgender men took part in the online survey. Their age ranged from 22 to 43 years with an average of approximately 33 years (see Table 1). The distribution of educational levels is skewed, predominantly representing a highly educated sample. Most participants reported a monthly income between 6,000 and 10,000 Swiss Francs, positioning the majority as average to above-average earners, compared to the Swiss median monthly salary of 6,788 Swiss Francs in 2022 (71). The most frequent admission ICD-10 diagnoses were depressive disorder (F32, F33) (*n* = 31) and adjustment disorder (F43.2) (*n* = 34). Additionally, 31 individuals were diagnosed with diseases related to pregnancy, childbirth and puerperium (O99.3). Before completing the questionnaire, participants attended between 1 and 4 psychotherapy sessions with a psychological or psychiatric psychotherapist specialized in reproductive mental health. Additionally, 47% participants had previously undergone psychotherapy prior to their referral to reproductive mental health services (see Table 2). There were no significant differences in stigma for patients who attended more than 5 psychotherapy sessions with reproductive mental health services or had previously undergone psychotherapy compared to those who did not.

**Table 1:** Sociodemographic characteristics of participants.

Characteristics	*n*	N/A	(%)	M	(SD)	Mdn
Age	109	13		32.85	(4.83)	33.00
18–29	27		(22.1)			
30–39	73		(59.8)			
40–49	9		(7.4)			
Gender	109	13				
Female	106		(97.2)			
Male	3		(2.8)			
Transgender	0		(0.0)			
Non-binary	0		(0.0)			
Education (highest degree)	109	13				
No qualifications	1		(0.8)			
Compulsory education	6		(4.9)			
Upper secondary education	41		(33.6)			
Tertiary education	61		(50.0)			
Income (monthly)	101	21				
Up to 4'000 CHF	13		(10.7)			
4'000–6'000 CHF	17		(13.9)			
6'000–10'000 CHF	49		(40.2)			
10'000–16'000 CHF	21		(17.2)			
More than 16'000 CHF	1		(0.8)			

**Table 2:** Clinical characteristics of participants.

Characteristics	*n*	N/A	(%)
Number of therapy in specialized outpatient service	93	29	
1	41		(33.6)
2	23		(18.9)
3	17		(13.9)
4	5		(4.1)
5+	7		(5.7)
Number of diagnoses at admission^a^^,^^b^			
F10 Mental and behavioral disorders due to psychoactive substance use	2^b^		
F20 Schizophrenia, schizotypal and delusional disorders	1		
F31 Bipolar affective disorder	1		
F32 Depressive episode	17		
F33 Recurrent depressive disorder	14		
F41 Other than phobic anxiety disorders	8		
F42 Obsessive-compulsive disorder	3		
F43.1 Post-traumatic stress disorder	2		
F43.2 Adjustment disorder	34		
F60 Specific personality disorders	7		
F90 Hyperkinetic disorders	5		
O99 Other obstetric conditions, not elsewhere classified	31		

a According to World Health Organization. (2004). ICD-10: international statistical classification of diseases and related health problems: tenth revision, 2nd ed. World Health Organization.

b Only absolute numbers shown because participants could have multiple diagnoses.

### 2.4 Procedure

Between October 2023 and December 2024, 374 patients attended an initial consultation in reproductive mental health services. Of these, 34 were excluded due to insufficient German language skills (*n* = 16), lack of a clear indication for therapy (*n* = 9), missing email contact details (*n* = 5) or because the consultation did not qualify as an initial appointment (*n* = 4). The remaining 340 received a study flyer by their therapist and were then were contacted by the study team via email, which included detailed study information and a personalized survey link. The survey link entailed a detailed information about the study including contact details for further questions and clarifications. Participants were informed that data was collected anonymously and that there will be no negative consequences if they decided not to participate in the study and they were able to stop participation at any time. The survey could only be started if participants gave informed consent after reading the study information. The survey took about 20–30 min to complete. Non-respondents received up to three weekly reminders via email and SMS. Patients who did not complete the survey within one week of the final reminder or who declined participation were excluded. In total, 206 individuals did not respond or declined to participate. 12 further participants were excluded because they withheld consent. Data from 122 participants who completed the survey after giving informed consent were included in the final analysis, corresponding to a response rate of 32.6% of all 374 patients who attended at least one psychotherapy session.

### 2.5 Measures

#### 2.5.1 Self-stigmatization

Self-stigmatization was assessed using the German version of the Internalized Stigma of Mental Illness Inventory (ISMI-29) (32, 72) with 29 items rated on a four-point Likert scale ranging from 1 = “strongly disagree” to 4 = “strongly agree”. We excluded the Stigma Resistance subscale because several studies have shown that it does not act as a significantly independent predictor of self-stigma. The Stigma Resistance subscale stands out due to its psychometric weaknesses as well as its conceptual ambiguity. In terms of content, this subscale appears to capture coping strategies and resilience rather than internalized stigma itself and therefore does not fit well into the overall concept of internalized stigma. (Brohan et al., 2010; Lysaker et al., 2007, 32). The ISMI-29 shows high internal consistencies in the German (*α* = 0.92) (32) and English version (*α* = 0.90) (73). Mean scores were used for the analyses.

#### 2.5.2 Process of seeking therapy

To examine what may have led patients to start attending reproductive mental health services we used the four steps of help seeking: (1) realizing there is a problem, (2) deciding that therapy would be an appropriate way to try to solve the problem, (3) deciding to seek therapy, (4) making contact with the mental health system (48). Accordingly, participants have been asked the following questions: (1) “How long has it been since you first thought that you might be suffering from psychological issues?”, (2) “How long has it been since you first considered that psychotherapy might help with your current issues?”, (3) “How long has it been since you first thought about seeking psychotherapy for your current issues?”, (4) “How much time has passed since you first contacted a professional to schedule a psychotherapy appointment?”. To facilitate the conversion process, participants could report their response in weeks, months, or years. For the analyses, the time unit was processed in weeks.

#### 2.5.3 Experiences with primary and reproductive healthcare professionals

For the quantitative analyses, participants were asked which of the following professionals had recommended or informed them about available psychotherapeutic treatments: gynecologist, midwife, doula, social educator, psychologist, none, or other. The total number of marked professional groups and/or additional professional groups added under “other” constituted the sum of involved professionals. Additionally, the participants were asked which of these professionals they would like to be approached by regarding their psychological issues, which they explicitly would not want to approach them, and from whom they would like to receive specific information about treatment options.

In the qualitative part of the study, participants were asked the following four questions: (1) “If you were approached by the aforementioned professionals about psychological issues: What was helpful/motivating?”, (2) “If you were approached by the aforementioned professionals about psychological issues: What was discouraging/demotivating?”, (3) “Which behaviors/characteristics of the professionals have you found particularly valuable?”, (4) “What additional or different actions would you have hoped for from professionals?”.

#### 2.5.4 Expectations concerning psychotherapy

To examine their expectation and concerns around psychotherapy, the following two open-ended questions were asked: (1) “What are your expectations from psychotherapy in reproductive mental health services?” and (2) “What concerns do you have regarding psychotherapy in reproductive mental health services?”.

### 2.6 Data analysis

#### 2.6.1 Quantitative analysis

Statistical analyses were performed using IBM SPSS Statistics (IBM SPSS, 2024). We tested H1a-H1d with linear regressions and H2 using a t-test.

Data were screened for outliers using the 1.5 IQR method. For regression models, cases were excluded if therapy-seeking durations exceeded the upper IQR bound (IQR × 1.5) or three years (156 weeks), as these were considered non-peripartum trajectories. For self-stigma scores, three outliers were identified but retained due to consistent response patterns. Sensitivity analyses showed that excluding these cases did not alter the results.

#### 2.6.2 Qualitative analysis

The qualitative data was extracted from the online survey into a word document; data coding was done in MAXQDA. The data was organized according to the two main topics to which participants responded: 1) experiences with general and reproductive healthcare professionals and 2) expectations concerning psychotherapy.

Data was analyzed with thematic analysis, using an inductive-semantic approach, as we wanted to learn about experiences, feelings, thoughts of participants on their way to seek treatment and in respect to the treatment itself. This was combined with a deductive approach, that focused on motivating/helpful and demotivating factors on the way to seek treatment and positive and deterring treatment expectations and aspects of experienced stigma or self-stigma in the data. Data analysis was conducted by Katrin Braune-Krickau and Anna-Lena Horn and guided by the six steps outlined by Braun and Clark (74, 75).

Although distinct themes could be identified, their definitions often remained at a descriptive level, as many participants provided one-word or bullet-point responses.

##### 2.6.2.1 Subjective position of researchers

Katrin Braune-Krickau is a researcher and child psychotherapist specializing in infant mental health. She provides parent-infant psychotherapy and has extensive experience working closely with gynecopsychiatric services to support families in which a parent suffers from a postpartum disorder. In her clinical work with older children and adolescents, she frequently collects family histories and has heard numerous accounts of parents who experienced postpartum depression that went unrecognized and untreated. These parents often feel that this had long-lasting consequences for the parent-child relationship.

Anna-Lena Horn recently completed her master's degree in applied psychology. With an initial professional background as a childcare specialist, she has many years of experience working pedagogically with children and adolescents. She currently works as a psychologist in a child and adolescent psychiatry setting, where she repeatedly encounters parents who are open to receiving support but find accessing psychotherapy difficult and overwhelming. Through interdisciplinary exchanges with professionals, she has also observed a shared uncertainty about how to better support parents in seeking and receiving early and appropriate psychotherapeutic treatment.

Katrin Braune-Krickau and Anna-Lena Horn share the conviction that supportive, need-oriented, and specialized psychiatric and psychotherapeutic treatment for parents in the peripartum period benefits not only the patient but the entire family—especially the infant's development. Due to their professional backgrounds, they have a positive bias toward general practitioner (GP) treatment. Both are motivated to learn from the data how to improve access to necessary services for parents and to gain insights into how professionals can help pave the way to treatment.

## 3 Quantitative results

### 3.1 Associations between self-stigma and help-seeking behavior

Descriptive analyses revealed a relatively low overall level of self-stigma in the sample (M = 1.57, SD = 0.45, range = 1.00–2.75). Overall, half of the studied individuals took over 2 years for completing all 4 steps (48). The durations reported for each step of the therapy-seeking process were highly variable, with mean values of weeks decreasing across the four steps: recognizing a mental health problem (M = 106.05 weeks, SD = 192.21), considering psychotherapy as a suitable option (M = 73.95 weeks, SD = 150.44), deciding to seek therapy (M = 53.78 weeks, SD = 127.44), and initiating contact with a mental health service (M = 19.26 weeks, SD = 46.53). Despite these considerable delays, linear regression analyses showed no statistically significant associations between levels of self-stigma and the duration of each of the four steps: recognizing a mental health problem [F(59) = 0.033, *p* = 0.856], considering psychotherapy as a suitable option [F(65) = 0.281, *p* = 0.598], deciding to seek therapy [F(67) = 0.063, *p* = 0.802], deciding to seek therapy [F(73) = 0.048, *p* = 0.828]. See Table 3 for further statistics on these regression analyses. Thus, our data do not support the assumption that higher levels of self-stigma are linked to longer delays in the process of seeking therapy.

**Table 3:** Descriptive statistics of stigma and steps of the process to seeking therapy.

Variable	*n*	N/A	M	(SD)	Mdn	Min	Max
Self-Stigma total score	78	44	1.57	0.45	1.46	1.00	2.75
Steps of the process to seeking therapy in weeks							
Step 1: realizing there is a problem	87	35	106.05	192.21	24.00	1	1,040
Step 2: deciding that therapy would be an appropriate way to try to solve the problem	91	31	73.95	150.44	12.00	1	936
Step 3: deciding to seek therapy	89	33	53.78	127.44	8.00	1	936
Step 4: making contact with the mental health service	92	30	19.26	46.53	4.00	0	312

A comparison of participants who reported being approached by professionals (77%, *n* = 60) and those who were not (21%, *n* = 16) revealed no statistically significant difference in self-stigma scores in the independent samples t-test, t(74) = –0.11, *p* = 0.46, with a small effect size (Cohen's d = 0.03). Participants who had been approached reported a mean self-stigma score of M = 1.55 (SD = 0.45), while those who had not been approached had a mean score of M = 1.56 (SD = 0.47). Based on these results, our hypotheses were not supported and were therefore rejected (see Table 4).

**Table 4:** Linear regression of self-stigma on the steps of the process of seeking therapy.

Steps of therapy-seeking-process	*n*	*b*	SE	*β*	*t*	*p*	Korr. R^2^
1: realizing there is a problem	61	−2.693	14.779	−0.024	−0.182	.856	−0.02
2: dividing that therapy would be an appropriate way to try to solve the problem	67	4.189	7.897	0.066	0.530	.598	−0.01
3: deciding to seek therapy	69	−2.234	8.884	−0.031	−0.251	.802	−0.01
4: making contact with the mental health service	75	1.272	5.821	0.026	0.219	.828	−0.01

Step 1: F(59) = 0.033; Step 2: F(65) = 0.281; Step 3: F(67) = 0.063; Step 4: F(73) = 0.048.

### 3.2 Preferences for professional contact in the help-seeking process

**Figure 1:**
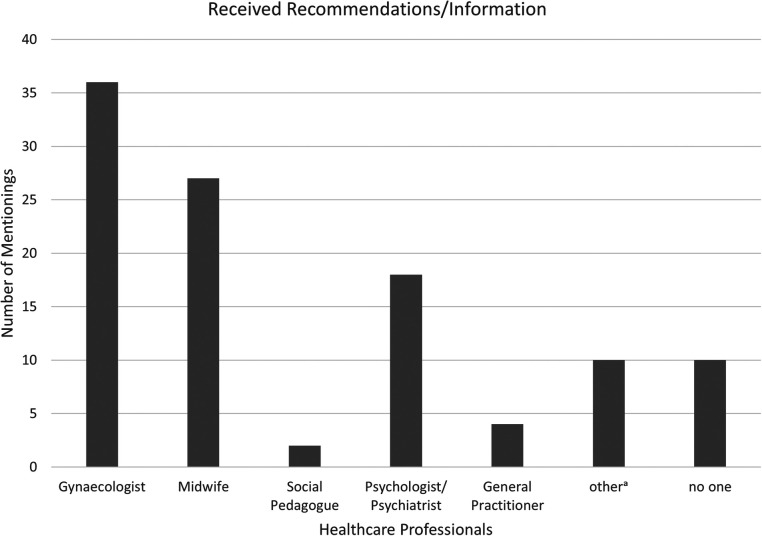
Number of healthcare professionals that gave recommendations/information. ^a^myself (3), social environment (3), internet (1), parent counselling (2), kinesiologist (1).

Participants were asked which healthcare professionals had provided them with a recommendation for psychotherapy or shared information about available mental health services. As shown in Figure 1, more than one third of participants reported receiving such a recommendation from their gynecologist, while slightly fewer reported receiving it from a midwife. Those who had previously been in psychological or psychiatric treatment most commonly cited psychologists or psychiatrists as the source. A few participants also mentioned their GP.

**Figure 2:**
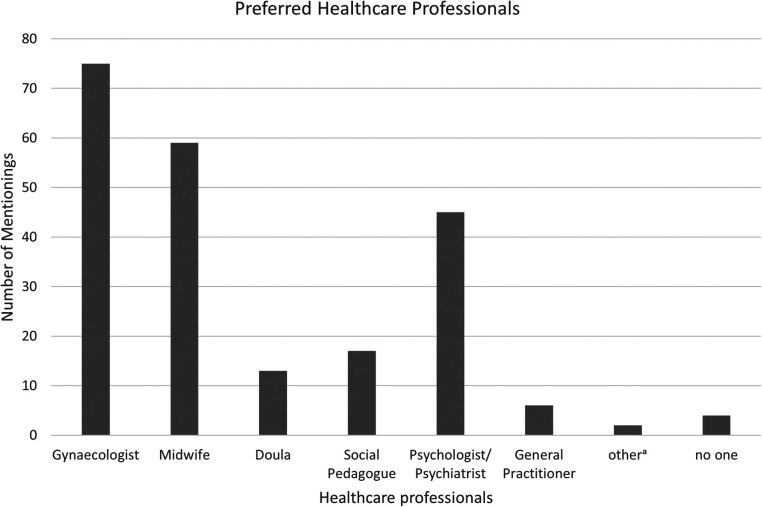
**Preferred health care professionals for addressing mental health concerns.** Number of preferred healthcare professionals for addressing mental health concerns. ^a^All healthcare professionals (1), fertility clinic (1).

In a separate item, participants indicated their preferences regarding mental health conversations. As illustrated in Figure 2, more than two thirds of the respondents stated that they would like to be approached about mental health concerns by their gynecologist, and well over half expressed the same preference for midwives.

Only 11% of the participants explicitly stated that they did not wish to be approached by specific professional groups. These included gynecologists (*n* = 3), midwives (*n* = 1), doulas (*n* = 2), and social workers (*n* = 2). The remaining 89% of participants did not select any group they would prefer not to be approached by, suggesting that most were in principle open to being addressed about mental health concerns—though preferences vary.

A similar pattern emerged regarding the desire for concrete information on where to find help for psychological distress. Again, participants most frequently indicated gynecologists, midwives, and psychologists as the professionals from whom they would most like to receive such information.

## 4 Qualitative results

### 4.1 Results from domain 1: experiences with primary and reproductive healthcare professionals

Participants were asked to describe behaviors or comments from professionals that either motivated and encouraged them to seek help in the peripartum specialist psychiatric service or deterred them from doing so, but many answers also described more general experiences with professionals. The following is a detailed description of the eight identified themes with corresponding examples from the data collection, a tabular overview of the themes can be found in the Supplementary Materials.

#### 4.1.1 Overarching theme 1. Not beating around the bush –clear words instead of overlooking or downplaying psychological distress

This overarching theme unites two themes that address how professionals communicate about participants' experiences of distress, worries, and feelings. On one hand, participants found it helpful when professionals directly addressed their situation (e.g., emotions, thoughts, states of distress, anxiety, or depression.) This direct approach enabled them to acknowledge their need for help and take the necessary steps to actively seek it. On the other hand, failing to address the situation directly—or downplaying it—was described as a behavior that deterred participants from seeking help.

##### 4.1.1.1 Theme 1.1. Ignoring and dismissing

This theme describes experiences in which an emotional or psychological state of distress was either completely ignored by professionals or addressed in a manner that felt dismissive, leaving participants feeling that they were not taken seriously. For example, while many participants noted that none of the professionals addressed their distress, one participant remarked that it was demotivating “if a topic isn't addressed directly or if people beat around the bush” (P1005.). Other participants highlighted the negative impact of receiving platitudes such as being told that it is normal to feel exhausted when having children or having their concerns minimized as merely a hormonal issue (P1170.).

##### 4.1.1.2 Theme 1.2. Clear words and acknowledgment of distress

Participants experienced open and clear communication from professionals as positive. One participant stated, “open communication was very important for me” (P1105.) The interviewed participants appreciated not only being asked about their emotional state or distress but also described the relief through acknowledgment of the seriousness of the situation by a professional. Participant 1,170 explained: “Knowing that something is not okay (through an encounter with a professional)—you keep thinking/telling yourself, it's not that bad and maybe you experience it as worse than it really is.” This comment speaks to the self-expectation of having to struggle through it, but also to a feeling of not completely trusting one's own appraisal of distress. External confirmation seems to be helpful in getting participants out of a zone of doubt about their own psychological state. Another participant described receiving external confirmation of the extent of psychological distress when completing a self-test with a midwife: “There was a self-test that I filled out with my midwife, and it showed very high scores.” (P1367.)

#### 4.1.2 Overarching theme 2. Wanting to feel understood and accepted as opposed to condemned, judged, and devalued

This overarching theme connects the themes *being judged, especially as a mother* as a negative experience in interactions with professionals before seeking help and *being accepted and understood without judgment* as positive and motivating experiences in encounters with professionals. These themes are closely tied to the topic of stigma and describe the negative and positive poles of moral judgment from others.

##### 4.1.2.1 Theme 2.1 being judged, especially as a mother

This theme reflects the experience of feeling judged for one's emotions, fears, or overall psychological distress—often associated with peripartum psychiatric disorders or other issues for which participants sought help. A critical or judgmental attitude by professionals was seen as demotivating. One participant specifically mentioned the fear of being condemned as a mother or being perceived as incompetent: “Being judged/condemned (especially as a mother); not being “good’ enough or competent enough.” (P1078.) This answer describes a core fear many women suffering from peripartum psychological disorders experience, the fear that they are failing at being a good mother. While participant 1,078 described this as a fear and not an actual experience, participant 1,076 described that she felt devalued in an actual encounter with a professional (her gynecologist during a pregnancy checkup) in the following sentences: “Furthermore, she said that I should have thought about that before when I revealed my slight worries and fears. I think any pregnant woman can feel overwhelmed and wonder how it will be with the new life… then I regretted having told the gynecologist about my problems!”

##### 4.1.2.2 Theme 2.2. Being accepted and understood without judgment

It was experienced as helpful by participants if professionals conveyed to them that whatever they felt and thought was o.k., that there was nothing wrong with that. The participants appreciated an open-minded, non-judgmental attitude toward their thoughts, feelings, and psychological distress. Additionally, for participants feeling understood was very important, this could be expressed by words, facial expressions or an empathic stance and it was very helpful if the professionals also showed their understanding trough body language.

##### 4.1.2.3 Theme 3. Others have this too – normalizing

Participants seemed to feel relieved through reassurance by professionals that what they experienced was normal and their symptoms quite common. This points to the underlying fear of not being normal, which describes the experience of many parents with postpartum depression or other post- or prepartum disorders. Unlike the *Ignoring and Dismissing* theme, where experiences were overlooked or played down, this theme highlights the positive impact of normalizing these experiences. Learning that this disconcerting or even disturbing experience is not monstruous, but shared by many other parents was comforting. Participant 1,262 has described not feeling so isolated anymore through “Knowing that you are not alone and that other women also need support.” These answers and additional remarks seem to mostly refer to the experience of postpartum depression and indicate that participants experience it as something that puts them outside of feeling normal and being seen as normal, an aspect that is relevant to stigma.

##### 4.1.2.4 Theme 4. Making sense – there are explanations and reasons for my condition

Additionally, to the theme of *normalizing* the experience of postpartum psychological disorders, the theme of learning that this distressing and disconcerting experience can be made sense of with the help of professionals seems to provide a sense of relieve to participants as well. Participants found it helpful to learn about reasons and explanations for their psychological state. One participant described an explanation she was given for her postpartum depression as follows: “That a postpartum depression could almost be inevitable given my history.. [number of children, various birth complications, premature birth, genetic predisposition, and my own psychiatric history].” (P1157.) Talking to professionals enabled some of the participants to better understand themselves and their experiences, as well as the underlying reasons for their distress and to look at their situation from a different perspective. Participant1079 mentioned that it was helpful to get “Explanations/assumptions about what it is all about.” from professionals. Another participant stressed that talking to a professional allowed her to take a new perspective on her situation: “It was helpful that I could look at some things from another perspective.” (P1274.) Knowing about the background of a potentially confusing and distressing experience might provide a feeling of agency toward this experience, of being less at the mercy of something that is unknown.

##### 4.1.2.5 Theme 5. Encouragement and hope: there is the right help for this

Participants felt reassured and supported by learning from professionals that specific help (referring to the reproductive mental health services) was available for them and that solutions existed that were tailored to their problems. The perspective of a solution, of a specialized solution for one's problems seems to counteract a state of feeling overwhelmed and to put participants into a position of becoming active in addressing their need for support. One participant described this in the following words “That there are people who are professionally trained in this area and can help you better.” (P1097.) Several participants also mentioned that they experienced encouragement from professionals to access peripartum services. One woman noted that the encouragement provided by professionals was instrumental in her decision to seek peripartum services. The hope that “things can get better” (P1128) reassured participants that they did not have to face their problems alone, as one woman put it: “She encouraged me to approach the psychologist and said that it would be supportive for me and my unborn baby.” (P241.) Another woman described that a professional recommended her to “Accept help without hesitation” she continued that it was helpful to “know that one can be healed of the distress.” (P1002.).

##### 4.1.2.6 Theme 6. Should I refer you right now/sign you up right now? Lowering the threshold while still leaving room for autonomy

An important topic for many participants was the referral process and how specialized reproductive mental health service could be accessed. This theme has a positive (supportive experiences with professionals) and a negative (unsupportive experiences with professionals) pole. Participants described it as helpful to receive clear information about the reproductive mental health service and how to access it. They appreciated a hands-on approach from professionals regarding referrals. This is reflected in this participant's answer: “The professional organized the first contact/session for me.” (P1276.) An uncomplicated stance with a low threshold access to GP service was appreciated, while some participants stressed that they valued having the autonomy to make their own decisions, as this participant commented: “the decision stays on my side.” (P1270.) Conversely, some participants missed receiving information, adequate support or guidance on how to register for treatment. Since postpartum depression or other postpartum disorders can go along with lack of energy, incapacity to be active, etc. it may be important that professionals actively help taking this first important step toward accessing treatment, without taking decisions completely out of the concerned person's hands.

### 4.2 Expectations concerning psychotherapy

Participants were asked to describe fears and hopes regarding treatment in gynecological psychotherapy before they started the treatment. The following is a detailed description of the eight identified themes with corresponding examples from the data collection, a tabular overview of the themes can be found in the Supplementary Material.

#### 4.2.1 Overarching theme 1. Perceptions of mental health services

In this overarching theme, the themes *What do people think?* and *Uncertainty* are combined. Both themes describe the fear of not knowing what to expect in psychotherapy, and concerns about how one's social environment will react to seeking psychotherapy.

##### 4.2.1.1 Theme 1.1. What do people think? – The fear of judgment

This theme describes the concerns of the participants about the reaction of one's social environment when seeking therapy. Both the reactions from close family members and societal prejudices are described by participants as obstacles in the process of seeking therapy. The participants describe the fear of being labelled, judged, and confronted with the stigma of mental illness. Participant 1,225 reported that her fear of judgment prevented her from reaching out for help. Another participant 1,347 expressed hesitation about undergoing psychotherapy because she was afraid “of being labeled as a bad mother”.

##### 4.2.1.2 Theme 1.2. Uncertainty –Is psychotherapy only about diagnoses and medications?

The participants reported experiencing uncertainty when seeking psychotherapy, describing it as “not knowing what to expect.” (1,262.) This theme highlights the lack of clarity regarding what psychotherapy entails and what it includes. The participants expressed concerns about whether therapy is the right choice, whether it will help, and whether their expectations can be met. For example, participant 1,241 stated: “I was a bit worried about whether this would help at all and how such a conversation with a stranger would go, whether I would even be able to build trust.” The main concerns expressed by participants revolve around diagnoses and prescribed medication. This uncertainty can be a barrier to seeking therapy, as another participant 1,293 described feeling discouraged “when psychotherapy is about psychotropic drugs and diagnosis.”

#### 4.2.2 Overarching theme 2. The desire for improvement in condition – benefits of psychotherapy

This overarching theme combines the two themes: p*rocessing and closure of past experiences* and *What can I do?* At its core, all the themes reflect the participants' wish for symptom improvement. They see psychotherapy as an opportunity to experience again more positive emotions, such as joy.

##### 4.2.2.1 Theme 2.1. Processing and closure of past experiences

Respondents see psychotherapy as a chance to process and bring closure to their past experiences. This theme centers on a forward-looking perspective, with participants hoping to move on from past events and regain a positive outlook on the future. This is exemplified by the statement participant 1,016: “That I can put my traumatic birth experience/end of pregnancy into a box and be ready for a second child.”

##### 4.2.2.2 Theme 2.2. What can I do? –Learning how to cope with symptoms

This theme reflects the respondent's hope of learning trough psychotherapy how to cope with their symptoms. For example, one participant expressed their desire to acquire the following in psychotherapy: “Some tools to process the trauma and to be able to see the positive when looking back.” (P1179.) Additionally, participants reported that they wish to learn strategies for managing their emotions. This is illustrated by the wish of another participant 1,057: “Help in dealing with my feelings (frustration, anger, helplessness, despair, envy.)”

##### 4.2.2.3 Theme 3. What is happening to me? – Knowledge helps to understand myself and my symptoms

The respondents expressed a desire to better understand themselves and their situation. This includes gaining knowledge about handling their child as well as receiving psychoeducation about their own mental health. By doing so, they are better able to recognize and understand their symptoms. Participant 1,065 sees psychotherapy as an opportunity: “So that I can better understand my thoughts.” Similarly, participant 1,081 hopes that therapy will provide “a central point of contact for everything related to pregnancy, the baby, what it does to me, what it does to my partner, etc.”

##### 4.2.2.4 Theme 4. I want to be a good mother and enjoy parenthood – The pursuit of being a good mother

This theme focuses on the respondents’ hope of being a good mother despite experiencing mental health challenges while also meeting personal and societal expectations. It highlights the fear of judgment and the underlying fear of not being a “good enough” mother. The participants report the hope for psychotherapy to support them in the transition into motherhood as can be seen in the following statement from participant 1,331: “There are many things that my subconscious has not yet processed. And I don't want this decision to have a negative impact on future motherhood.” Additionally, the following statement from participant 1,136 illustrates that beyond the motherly role, there is also a desire to strengthen both the partnership and personal well-being: “Find out how to happily balance work, children, a romantic relationship, and household responsibilities.”

##### 4.2.2.5 Theme 5. The desire for a safe point of contact – Support, open conversations and being heard

The focus of this theme is the desire to find a safe point of contact through psychotherapy. The respondents find it supportive to have a consistent person to turn to. A central point mentioned by the participants is that “someone accompanies them through the process who understands them” (P1331). Furthermore, the respondents express the wish to be able to speak openly in psychotherapy. Participant 1,262, for example, sees it as an opportunity to “talk about fears, grief, and experiences with a neutral person.”

##### 4.2.2.6 Theme 6. Specialized focus in reproductive mental health services provides reassurance

This theme highlights the importance of a specialized focus on reproductive mental health. Respondents hope to receive guidance on specific topics such as “support with the desire to have children, pregnancy, and my depression” (P1045) or “support in dealing with miscarriage.” (P1109) The specialization was perceived as relieving by the participants and provided them with a sense of security.

## 5 Discussion

In this study we examined a sample of patients recently attending their first psychotherapy session(s) in a specialized outpatient service for reproductive mental disorders. This clinical sample of patients offers insights from those who did make it to psychotherapy despite existing barriers such as stigma or lack of knowledge. While such a sample bears a unique opportunity for understanding the way to psychotherapy in everyday clinical practice, it is simultaneously a selective sample of patients who completed the help-seeking process successfully *and* agreed to taking part in a research study. Therefore, it is not surprising that the overall level of self-stigma was relatively low in this sample. Since a first contact with a psychotherapist took place, a process of de-stigmatization may have already happened by the time participants answered the online questionnaire. These might be some of the reasons why the quantitative analyses did not show any significant association between self-stigma and the delays in the four steps of help-seeking. Additionally, there was a large range of weeks passed for each step in the sample. The average time of 106 weeks until recognizing their problem (step 1) can be viewed as a considerably large period of time – especially for those patients with young children and/or newborn babies. Therefore, we consider the examination of factors that may have contributed to acknowledging the problem and considering psychotherapy as clinically relevant for this and similar samples. Both the exploratory as well as the quantitative analyses shed light into possible factors that may help patients with reproductive mental disorders to get treatment faster.

### 5.1 Approaching mental health problems by professionals

In our sample, most patients were approached by at least one primary or reproductive healthcare professional concerning their mental health. The vast majority did not name a specific profession by which they did *not* want to be approached. To the contrary, 90% of the sample were open to discuss their mental health with different primary or reproductive healthcare such as midwifes, gynecologists or GPs. This indicates that those affected by mental disorders are open to being approached by primary or reproductive healthcare professionals to talk about their mental health.

In most cases conversations about mental health were initiated by gynecologists, midwives or previously consulted psychotherapists. These may therefore be important interfaces for the treatment of reproductive mental disorders. This is in line with recommendations made by the Lancet (Europe) stating: “To shift from stigmatizing perinatal mental health conditions to supporting mothers will require multidisciplinary approaches for early identification and prompt intervention. Collaborative action is imperative, requiring the commitment of clinicians, health-care providers, policy makers, and communities to prioritize maternal mental health within reproductive healthcare” [(49), p. 2].

However, primary or reproductive healthcare professionals are often not well trained in discussing mental health with their clients. A review showed that midwives “require continuous professional development opportunities that address knowledge, […], communication and assessment skills” concerning peripartum mental health [(50), p. 2]. Similar gaps in training were found in obstetrics and gynecologists (51, 52). Therefore, training mental health literacy in primary or reproductive healthcare professionals may be an important contribution to a better provision of mental healthcare in patients with reproductive mental disorders.

### 5.2 De-stigmatization through primary or reproductive healthcare professionals

In order to approach the topic of mental health there are specific recommendations that can be made based on the open-ended answers of this sample. The examination of their experiences with professionals revealed two major themes. First, participants did not want professionals to “beat around the bush”, as this was often experienced as being ignored or dismissed. Rather, they profited from clear word and acknowledgement of their problems. This is line with existing research on training healthcare providers to talk about mental disorders. For example, it is recommended to explicitly use medical terminology for mental disorders, as that also supports the legitimacy of mental illness and challenges the outdated stigma that “it's all in your head’ (53). Furthermore, explicit mentioning of mental disorders was associated with more positive attitudes toward mental health treatment seeking (54). Accordingly, one participant reported that it was important for them to know “that you are not alone and that other women also need support.”

Furthermore, talking about the existence of mental disorders in the reproductive and peripartum context may also lead the way for developing explanations as to what is happening and how one may get out of mental distress. Participants in this study were wishing for an explanation in order to “make sense” of their current mental state. Making sense of burdening mental states may be especially important for parents, among whom the stigma of mental disorders is often accentuated. For parents, mental illness stigma is often interconnected with perceived violations of social and cultural norms related to parenting (55). Such norms usually weigh especially heavily on mothers. Mothers were found to be more likely than fathers to perceive and internalize stigma linked with mental illness (56). Accordingly, participants in our study reported not wanting to be judged - especially as a mother. Hence, de-stigmatization may be especially important for mothers affected by mental disorders as well as their children, since stigma may even negatively affect parenting and, in turn, the development of their children (57).

Informing parents that mental disorders in the peripartum period affect many parents may contribute to destigmatization and can also increase the feeling of “not being alone” thereby creating a sense of connection between those affected. By having a name for what they experience, patients may also connect with others going through something similar. Accordingly, peer support is often an important additional factor for recovery from mental disorders (58).

### 5.3 Hopes and fears concerning psychotherapy

Once there is an open discussion about mental disorders in a de-stigmatizing way, there usually remains some hesitancy towards initiating psychotherapy. As mentioned above, the general public has generally a relatively poor knowledge of mental health symptoms and treatment (44). Such knowledge gaps combined with a long history of mal-treatment and exclusion of people affected by mental disorders (59) leaves room for mis-conception and fear of psychotherapy. Accordingly, participants reported a fear of psychiatric diagnoses and skepticism towards psychiatric medication as well as uncertainty about what psychotherapy entails. In line with techniques like motivational interviewing, change usually also entails disadvantages (60) and therefore, room should be given to counter-arguments for behavior change (in this case seeking psychotherapy). Talking about fears concerning mental health services may be an important corner stone for getting treatment.

Besides fears, hopes and needs are central to motivate change. Accordingly, the second overarching theme for patients was the desire for improvement in condition. Specifically, participants reported that “getting out of the depressive hole” and “overcoming fears” were motivating factors for starting psychotherapy. Furthermore, they were hoping to learn coping strategies and having guidance in processing past experiences. These hopes are all realistic goals for psychotherapeutic treatments and could be used to describe to parents what reproductive mental health services can offer. This could make the therapeutic process less threatening and help patients take the decision for treatment. After all, deciding that therapy might help seems to be the most difficult step (47). Furthermore, positive expectations of psychotherapy may even positively affect treatment outcomes (61).

Last, there were motivational factors that were specific to the context of reproductive mental health. Specifically, the availability of a specialized service such as reproductive mental health services was perceived as reassuring by the participants and provided them with a sense of security. Besides the overarching theme of “wanting to be a good mother” and “to enjoy parenthood”, specific requests were named such as “support with the desire to have children” or “support in dealing with miscarriage.”. Accordingly, people with reproductive or peripartum mental disorders may not only benefit from but also wish for services specialized to their needs.

### 5.4 Limitations

One major strength of the current study is also one of its biggest limitations. Since we used a clinical sample of patients newly attending the outpatient service for reproductive mental health, results are lacking generalizability. While this sample provides insight into those who did seek help in psychotherapy, we cannot make assumptions about those that did not. It is possible that patients with higher distress were less likely to participate. Thereby we may have missed information on the most vulnerable group of patients, since higher distress can be associated with more difficulty in deciding that therapy might help and deciding to seek therapy (47). This problem may be accentuated in men with mental disorders in peripartum, as research shows that the process of help-seeking tends to take even longer in men compared to women (62, 63). Hence, the low number of male patients may represent a deficit of psychotherapy admission in men, especially fathers. This is in line with the underrepresentation of men in the outpatient service according to the authors' clinical experience. Furthermore, at the time of data collection, menopausal mental disorders were not yet part of the specialized services described. However, menopause bears a similarly high risk for mental disorders as the peripartum period, but is still overlooked in many mental health services (64, 65). Therefore, mental disorders associated with menopause should be included in future studies as well as mental health services.

In addition, the relatively low response rate (32.6%) raises the possibility of a non-response bias. It is conceivable that those who were able and willing to complete the extensive questionnaire represent a subgroup of patients with comparatively greater psychological resources or lower burden, whereas individuals with more severe distress or less available capacity may have refrained from participating. This selective participation process also introduces a self-selection bias, as the decision to take part in the study was voluntary and may systematically favor patients who are more motivated, health-conscious, or less impaired. Consequently, our findings may reflect the perspectives of individuals with comparatively better coping resources, while underrepresenting those in more acute need of support. The same may be true for patients that did not fulfil inclusion criteria. Participants had to be able to speak and read the local language. Thereby, patients with refugee or migration experiences may have been underrepresented in this sample. This further limits generalizability, especially since the process of help-seeking may differ for different ethnicities or cultures (66).

Low generalizability may also be reflected in the asymmetrical distribution across several variables, which must be considered a limiting factor for the quantitative analyses. Furthermore, while the sample of 109 participants was considerably large for a clinical sample, the total sample size was well below the *a priori* calculated value of 200 participants, which would have been necessary for the calculation of significant results with medium effect sizes. In order to gain a deeper insight into a sample of patients who decided to start with psychotherapy we added qualitative analyses to the quantitative analyses. To keep the participation burden as low as possible, the data collection for the qualitative analysis was carried out with open-ended questions within the online questionnaire. While efficient, this method is likely to have resulted in less rich data than other collection methods such as semi-structured interviews for example.

### 5.5 Implications

As there is little literature to date with analyses of comparable samples, this study harbours potential for insight into successful help-seeking processes in patients with reproductive mental disorders. To gain a deeper understanding, future studies could include groups with and without mental disorders, who are not seeking psychotherapy (yet). Furthermore, semi-structured interviews with interpreters could be used to include patients who do not speak the local language.

Despite its limitations, the current study makes it possible to deduce various clinical implications. In Box 1, we formulated recommendations for primary or reproductive healthcare professionals based on findings of this study. In Box 2, recommendations for psychotherapists and providers of psychiatric services are formulated. Reproductive mental disorders are common and still often overlooked. Once detected, those affected face a myriad of hurdles such as hesitation or stigmatization in professionals, stigma concerning mental health as well as misconceptions about mental health services. Therefore, an open conversation and provision of information about mental disorders may help patients to overcome said hurdles and get the treatment they need as soon as possible.

Box 1 **Recommendations for primary or reproductive healthcare professionals.**

• Talk about mental health with all clients (don't beat around the bush)

• Acknowledge struggles (no downplaying or glossing over)

• Recognize mental disorders as common complaints (no judgement)

• Inform about special services and benefits of psychotherapy (encouragement)

• Provide support to get in touch with professionals (help getting help)

Box 2 **Recommendations for psychotherapists.**

• Explain how mental disorders occur (in this specific context)

• Have specialized knowledge (about this clientele)

• Help those affected to help themselves (coping)

• Give hope about possible changes (get out of the depressive hole)

• Support grieving process (past experiences like loss, birth and mental disorders)

## Data Availability

The raw data supporting the conclusions of this article will be made available by the authors, without undue reservation.
