# Supplementary material for: Access and barriers to reproductive mental health services. A mixed-methods examination of self-stigmatization, help-seeking motivation and experiences with primary or reproductive healthcare professionals
Source: Front Glob Womens Health. 2026 Jan 5;6:1658534. doi: 10.3389/fgwh.2025.1658534 (PMC12812951; doi:10.3389/fgwh.2025.1658534)
Supplement: Supplementary file 1 [file Table1.docx]

**Supplementary Materials**

**S 1. Overview of Themes and Codes: Encounters with professionals**

| Overarching Theme: “Not Beating Around the Bush – Clear Words Instead of Overlooking or Downplaying Psychological Distress” | |
| --- | --- |
| Theme: Ignoring and dismissing | |
|  | Not being taken seriously |
|  | Ignoring of feelings, not addressing topic |
|  | Nobody addressed it |
|  | Beating around the bush |
|  | Trivializing distress through professionals |
| Theme: Clear words and acknowledgment of distress | |
|  | Acknowledging the distress |
|  | Clear and direct communication (+) |
| Overarching theme: Wanting to feel understood and accepted as opposed to condemned, judged, and devalued | |
| Theme: Being judged, especially as a mother | |
|  | Judgmental attitude |
|  | Devaluation through professionals |
| Theme: Being accepted and understood without judgment | |
|  | Being accepted |
|  | Being understood |
| Theme: Others have this too/you are not alone – normalizing | |
|  | It’s normal |
|  | Others have this, too, you are not alone |
| Theme: Making sense – there are explanations and reasons for my condition | |
|  | New understanding and perspective of things |
|  | Understanding and making sense |
| Theme: Encouragement and hope: There is the right help for this | |
|  | The right support is out there |
|  | Encouragement to seek help |
|  | Not feeling alone anymore with the problem |
| Theme: Should I refer you right now/sign you up right now? Lowering the threshold while still leaving room for autonomy | |
|  | Room for autonomous decision |
|  | Professionals point the way to service, uncomplicated access |
|  | Information on treatment options |
|  | Referral directly through professional, short access route. |
|  | Support and advice |
|  | Learning about the specialized service from professionals |
|  | Helping to find a solution |

**Overview of Themes and Codes: Treatment expectations**

| Overarching Theme: Perceptions of psychotherapy | |
| --- | --- |
| Theme: What do people think – Fear of judgement | |
| Theme: Uncertainty – Is psychotherapy only about diagnoses and medication? | |
|  |  |
| Overarching theme: The desire for improvement in condition – Benefits of psychotherapy | |
| Theme: Getting out of the depressive hole and overcoming fears | |
| Theme: Processing and closure of past Experiences | |
| Theme: What can I do? - Learning how to cope with my symptoms | |
| Theme: What is happening to me? - Knowledge helps to understand myself and my symptoms | |
| Theme: I want to be a good mother and enjoy parenthood | |
| Theme: The desire for a safe point of contact – Support, open conversations and being heard | |
| Theme: Specialized focus in gyneco-psychiatry provides reassurance | |
